# Clinical Translation of Bio-Artificial Pancreas Therapies: Ethical, Legal and Psychosocial Interdisciplinary Considerations and Key Recommendations

**DOI:** 10.3389/ti.2023.11705

**Published:** 2023-09-18

**Authors:** Dide de Jongh, Rebecca L. Thom, Antonia J. Cronin, Eline M. Bunnik, Emma K. Massey

**Affiliations:** ^1^ Department of Nephrology and Transplantation, Erasmus MC, University Medical Centre Rotterdam, Rotterdam, Netherlands; ^2^ Department of Medical Ethics, Philosophy and History of Medicine, Erasmus MC, University Medical Centre Rotterdam, Rotterdam, Netherlands; ^3^ Guy’s and St. Thomas’ NHS Foundation Trust, London, United Kingdom; ^4^ King’s College, London, United Kingdom

**Keywords:** transplantation, regenerative medicine, tissue engineering, type 1 diabetes, informed consent

## Abstract

The field of regenerative medicine offers potential therapies for Type 1 Diabetes, whereby metabolically active cellular components are combined with synthetic medical devices. These therapies are sometimes referred to as “bioartificial pancreases.” For these emerging and rapidly developing therapies to be clinically translated to patients, researchers must overcome not just scientific hurdles, but also navigate complex legal, ethical and psychosocial issues. In this article, we first provide an introductory overview of the key legal, ethical and psychosocial considerations identified in the existing literature and identify areas where research is currently lacking. We then highlight two principal areas of concern in which these discrete disciplines significantly overlap: 1) individual autonomy and 2) access and equality. Using the example of beta-cell provenance, we demonstrate how, by harnessing an interdisciplinary approach we can address these key areas of concern. Moreover, we provide practical recommendations to researchers, clinicians, and policymakers which will help to facilitate the clinical translation of this cutting-edge technology for Type 1 Diabetes patients. Finally, we emphasize the importance of exploring patient perspectives to ensure their responsible and acceptable translation from bench to body.

## Introduction

The current mainstay of treatment for Type 1 Diabetes uses exogenous insulin administered either as intermittent injections multiple times a day, or through a continuous infusion pump. Unfortunately, these techniques cannot precisely mimic the function of the native pancreas and the regulation and administration of exogenous insulin can be a stressful and burdensome self-management task for patients [1]. For instance, they must continuously monitor dietary intake, physical activity, and resulting blood sugar levels, and to adjust insulin dosage when necessary [2]. Transplantation of either the whole pancreas or islet cells offers the potential to return the recipient to a more stable state of euglycemia without the need to administer insulin and prevent or delay the onset of diabetic complications [3]. However, both treatments are associated with significant risks such as complications of the procedure itself [4], increased propensity for infections and neoplasms as a result of immune suppression [5], and, in due course, graft failure [6, 7].

In addition, transplantation is not available for all patients with diabetes [8]. In low- and middle-income countries, transplant programs are limited by available health infrastructure. Even when islet and pancreas transplantation programs are established, the persistent global shortage of high-quality donor organs means that the availability of this therapy must be restricted [9]. Transplantation is reserved for a limited subgroup of patients with severe complications of diabetes, such as hypoglycaemic unawareness, suboptimal glycaemic control despite maximal medical input, and kidney failure [10]. Therefore, it is clearly imperative to develop alternative therapies, which help patients return to euglycemia without the limitations of existing transplantation options.

Regenerative medicine offers the most compelling prospects for developing such therapies. Regenerative medicine uses advanced biotechnologies, including tissue engineering and gene editing, to “replace or regenerate human cells, tissues or organs, to restore or establish normal function [11].” Research groups seeking to harness this technology to establish a novel treatment for diabetes have used a variety of biological and synthetic components. Insulin secreting cells from either deceased donors, xenogeneic cells or renewable induced allogenic stem cells are combined with other biological or synthetic (non-biological) devices such as scaffolds to hold or encapsulate the cells [12–23]. These products with both biological and device-based components can be seen as “hybrid” beta-cell replacement therapies and are sometimes referred to as “bioartificial pancreases.”

The prospect of a hybrid beta-cell replacement therapy for Type 1 Diabetes patients that is safe and effective is tantalizing. However, for such a therapy to be a successful alternative it must also overcome the limitations of existing therapies. Specifically, the ideal hybrid beta-cell replacement therapy for Type 1 Diabetes it would be:

• Personalised to reduce or remove the need for post-transplant immunosuppression
• Widely accessible and available for patients

If realized, this hybrid beta-cell replacement therapy could have a revolutionary effect on the management of Type 1 Diabetes world-wide. Even so, they are also uniquely complicated, not just from a scientific perspective but also from ethical, legal, and psychosocial perspective. While many of these issues in isolation are not novel, in combination they present a new level of complexity that is unique and challenging for researchers, clinicians and policymakers.

## Part 1—Ethical, Legal, and Psychosocial Overview

### Ethics

A recently published systematic review highlighted the ethical challenges of conducting early phase clinical trials of bioartificial organs [24]. Of most relevance to the clinical translation of this therapy for Type 1 Diabetes patients are 1) the source of the various cells used 2) recipient selection 3) informed consent and 4) access and justice considerations.

First, where hybrid products combine components made from cells and tissues (biomaterial) from different sources, each source (allogenic stem cells, deceased donor human islet cells and xenogeneic cells) will come with its own set of ethical considerations [24]. We explore this in more detail in Part 2 of this paper. Second, in the early phases of clinical translation, like with many novel therapies, not all Type 1 Diabetes patients may be eligible for hybrid beta-cell replacement therapy. This therapy requires an invasive and potentially irreversible surgical procedure, and the therapy may interact and integrate with the body, with unknown potential harms and complications [24]. Yet this is not immediately lifesaving. So, the balance of risks and benefits may not be favorable for Type 1 Diabetes patients who are relatively healthy. The first patients undergoing the therapy would likely be those who do not succeed in achieving adequate control of their glucose levels, suffer from the complications thereof, and have exhausted standard treatment options. Third, patient desperation for a cure can pose challenges to obtaining informed consent. Their desperation may lead to misunderstanding regarding the potential risks associated with participating in a clinical trial [24]. Uncertainties about complications arising from the novel nature of the therapy can hinder the provision of accurate information about the risk-benefit ratio of the intervention, this will also be explored in more detail in part 2 of this paper. Last, concerns regarding the accessibility of treatments for Type 1 Diabetes are not new nor specific to hybrid beta-cell replacement therapies, but these existing inequities may be amplified by this new technology. For example, currently some patients in developed countries cannot use the best available device-based treatments due to restrictive national reimbursement policies [25–27]. As with other regenerative medicine technologies, the costs of research and development of hybrid beta-cell replacement therapies will invariably be high. Nevertheless, access to these therapies should be equitable [8]. Ideally, they should be provided first and foremost to patients who stand to benefit the most.

### Law

As with the ethical issues, each new cell type or component included in the therapy brings with it legal and regulatory requirements, so that even within the European Union, this may result in a complex web of national and international regulatory instruments.

To demonstrate—when using human deceased donor pancreases as a beta-cell source, firstly national laws regarding organ donation must be adhered to. Organs which are donated are then subject to EU Directive 2010/45/EU on standards of quality and safety of human organs intended for transplantation [28]. Secondly, if beta-cells are extracted from the very same pancreas, but are cultured or manipulated, they may then be subject to directives on tissues and cells [29, 30] and/or genetically modified organisms [31, 32]. Thirdly, stem cells that have been gene-edited or induced may be subject to regulations on genetically modified organisms [31, 32]. Fourthly, supporting matrixes or scaffolds could be subject regulations governing tissues and cells [30] or medical devices depending on if they contain viable cells [33]. Fifthly, in the European Union a product such as this, which combines cells with devices, is likely to fall under the definition of an advanced therapy medicinal product (ATMP), which have additional specific regulatory requirements to adhere to (EU Regulation No 1394/2007) [34]. Thus, even with the advantages of harmonisation across the EU, there may still be multiple regulations or directives which are relevant to these products or their components throughout development and clinical translation.

What is more, there is not a global classification and many jurisdictions have taken alternative approaches, which has further increased the complexity of regulations and safeguards that developers must satisfy. Regulatory complexity and heterogeneity have been cited by legal academics and developers as a barrier to innovation even within single jurisdictions [35]. In response to such concerns, the World Health Organisation have issued a consultation urging for “Regulatory Convergence of Cell and Gene Therapy Products” to encourage research and enable broader access to such therapies [8].

In addition to these regulatory requirements, the areas of law which govern the everyday practice of clinical medicine, such as those relating to consent to medical therapy, confidentiality and equality of access must be considered. Taking the example of consent for medical treatment, this is protected at the highest level of European Law. The European Court of Human Rights (ECHR) has confirmed that Article 8 of the European Convention on Human Rights (ECHR)- the right to a private and family life- offers broad protection for individual autonomy [36, 37]. This includes the right to consent to, or refuse, medical treatment provided the person “is in a position to make up his own mind [38].” For consent to be considered valid, three principles are usually used—that it should be informed, given freely, and without coercion, and that the person should have the legal capacity to do so.

As hybrid therapies transition from investigational therapies to clinical practice informed consent could become a key factor in two ways. Firstly, the ability of potential recipients to understand the necessary information for such complex hybrid products has already been highlighted by researchers as a concern and is likely to be similarly problematic in clinical settings [24, 39]. Secondly, in contrast to research which is likely to only involve subjects able to consent, if a successful device moves into routine clinical care, it should also be available to those who have impaired capacity to consent, either by virtue of their age (minors) or due to impaired cognitive faculties. In each of these cases—determining the amount of information required for consent to be considered “valid” and the processes required to determine medical care in persons unable to give consent—the legal framework is decided at a national level [36] with differing practices, potentially resulting in differing availability across jurisdictions.

### Psychosocial

Whether new hybrid therapies succeed in improving the health and wellbeing of Type 1 Diabetes patients also depends societal context in which they are developed [40, 41]. Understanding of patients’ perspectives on hybrid therapies is important in order to ensure their responsible and acceptable translation from bench to body [42]. Yet there is a paucity of empirical research in this area [24]. It will be essential to explore the perceived advantages and disadvantages of hybrid therapies relative to treatment options currently available [42] as this is likely to impact uptake and adoption. For instance, while advanced device-based treatment options improve glycaemic control [43, 44] and make disease management easier [45], it remains challenging for patients to successfully learn how to handle these devices. For instance, several participants in a closed-loop system trial reported that they spent more time thinking about their diabetes while using this system than while undergoing standard treatment [1].

Another reported disadvantage of current treatments is the visibility of these devices due to having to wear a component on the body, such as a sensor on the arm. Some patients refrain from wearing pumps in public, to hide their disease from others, to preserve their self-image and to prevent stigmatization associated with having a disease [45]. An online survey study investigated the willingness of Type 1 Diabetes patients in the US to receive a personalized beta-cell replacement therapy as well as their preferences regarding the size, shape, visibility and transplantation site of the therapy. Findings suggested that the aesthetics are of importance to the majority of the patients surveyed [46]. There is also the implicit relationship between the human donors and recipients to be considered. For instance, some patients may have moral objections to having cells from a deceased person incorporated into the treatment [47], this will be further explored in part 2.

### Key Interdisciplinary Issues

**FIGURE 1:**
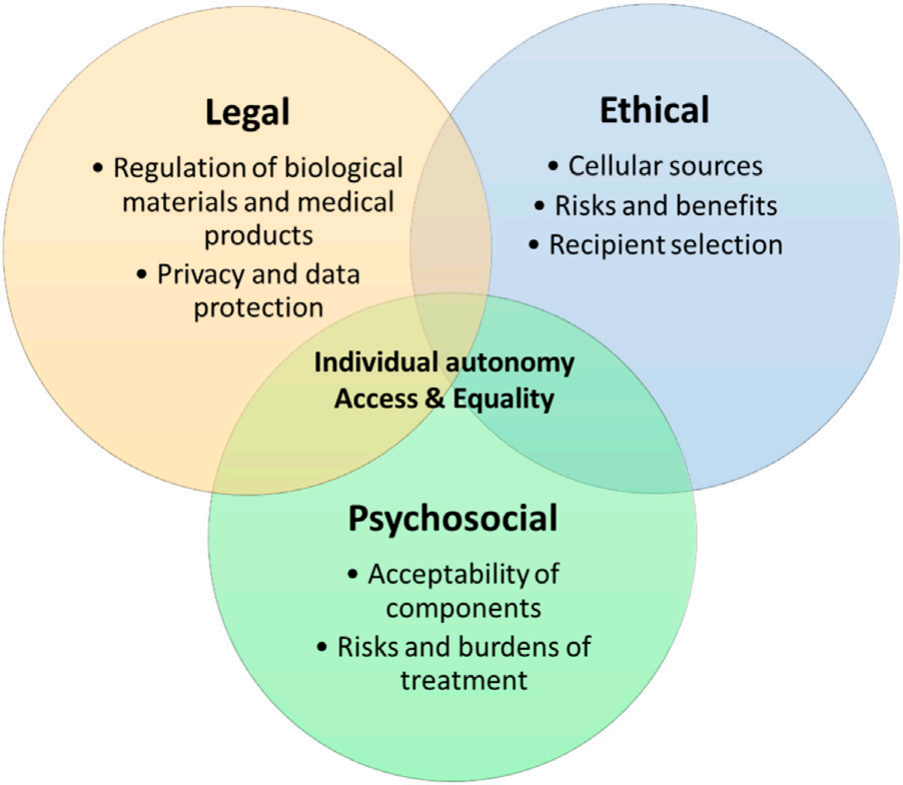
Overlapping ethical, legal and psychosocial issues.

In order for hybrid beta-cell replacement therapies to be clinically translated, these key ethical, legal, and psychosocial issues need addressing. However, these issues are not the domain of discrete disciplines, but are interwoven and must be addressed in conjunction to find successful solutions and this novel area of medicine to flourish (see Figure 1).

We set out two principal areas where these disciplines converge:

• Individual autonomy
• Access and equality

## Part 2—Examining Cell Source and Provenance Using an Interdisciplinary Lens

Issues related to the sourcing of the cells used to generate complex tissue-engineered products, such as bio-artificial organ, are the most frequently discussed aspects in the scientific literature [24]. To generate a hybrid beta-cell replacement therapy, a reliable and ideally renewable source (e.g., allogenic stem cells) of insulin secreting beta cells (see Table 1) must be identified. Each of these cell types will have scientific and practical advantages or disadvantages, but they also have distinctive ethical, legal and psychosocial features.

**TABLE 1:** Possible insulin secreting beta cell sources.

Allogenic stem cells
Deceased donor islet cells
Xenogeneic cells

Here, we examine the impact of cell source and provenance on the exercise of individual autonomy and on achieving equitable access to this novel therapy using an interdisciplinary lens.

### Individual Autonomy

The exercise of individual autonomy plays a significant role at multiple stages through the process of creating a therapy which utilises cells and tissues- in the act of donating biomaterials, the preferences and acceptability of cell sources to potential recipients and ultimately in gaining informed consent for the procedure.

#### Living and Deceased (Stem) Cell Donors

As outlined in the legal summary in the European Union, the acquisition, storage and use of human blood, cells, tissues, and organs is closely regulated. Most of these regulations are focused on the necessary conditions for procurement and testing of cells. Their purpose is to protect donors from exploitation and protecting recipients from risks such as transmission of infections or malignancies. However, the regulation preamble also hints at a philosophical purpose. It states:

“As a matter of principle, tissue and cell application programmes should be founded on the philosophy of voluntary and unpaid donation, anonymity of both donor and recipient, altruism of the donor and solidarity between donor and recipient [48].”

So, these regulations serve to protect both donors and recipients of allogenic cells, but also to promote a certain culture of altruistic and voluntary donation—an exercise of personal autonomy for the good of the community.

However, handling donor cells is not without ethical and psychosocial considerations. The collection and use of allogenic (stem) cells for clinical applications could raise concerns regarding the confidentiality and privacy of the donor, and on ownership and commodification of donated cells [47, 49–51]. Explicit informed consent from the cell donor or their family is required. As a prerequisite for donor consent, donors should be comprehensively informed on current and future cell usage, financial rights, policies regarding the return of findings and the option to withdraw [52]. Safeguarding donor privacy is essential, for example, achieved through anonymizing samples. However, achieving absolute anonymity of donor cells in the field of regenerative medicine is questionable for three reasons: 1) due to advancements in big data and genomics; 2) it is may not preferable as it hinders the return of results to donors and 3) unfavourable since donors lose control and the ability to manage their samples, including the option to withdraw. Recent empirical studies on tissue donation for organoid biobanking [53, 54] highlight that tissue donors desire information, control, and the ability to withdraw. Donors seek knowledge of research outcomes among recipients and the impact of the treatment, to ensure their contribution is meaningful. Their motivation to donate is most often rooted in the idea of beneficence to these unseen and unknown recipients. In addition, the question of ownership of collected cells and engineered tissues, involving the donor, the recipient and the producing parties, remains debated. When altruistically donated cells are turned into profitable products without financial compensation to cell donors, there is potential to violate human dignity and lead to exploitation.

From the perspective of recipients there could be moral and religious objections for the use of deceased donors or (decellularized) donor cells, which form psychological or social barriers to treatment [51]. For instance, some patients may argue that they do not want parts of another person to merge with their own cells. Recipients may create an image of, or develop a perceived bond with, the donor who provides the cells for treatment [47]. They may (or may not) struggle to accept that the cells that their treatment is dependent on are cells from a deceased person [47]. Or they may not wish to accept a therapy that contains genetically modified cells, because of the uncertainty of potential tumorigenicity or unwanted side effects. In addition, using modified cells in therapies for patients may portray the human body as malleable [51] and raise questions as to whether human cells should be subjected to engineering.

#### Xenogeneic Cells

Cells derived from genetically modified animal (namely, porcine) sources have been suggested as alternatives. However, the use of xenogeneic cells raises even more challenging potential psychosocial and ethical barriers leading to varying legal approaches. These include moral concerns for animal rights and welfare, religious beliefs [51, 55] and the risks for wider society, particularly that of zoonosis [51, 56]. The EU has issued guidelines for an approach to the medicinal use of animal cells [57]. However, due to the culturally sensitive nature of this topic the overall permissibility of xenotransplantation is a matter devolved to individual member states. Despite centralized recommendations, markedly different approaches have been adopted. For example, in Germany groups such as the Deutsches Primaten zentrum (German Primate Centers or DPZ) have been working extensively on xenotransplantation for over 20 years and are leading centers in porcine to non-human primate transplantation research. In contrast, the Netherlands have had a complete ban on xenotransplantation in place since 2002 [58]. Should a therapy containing animal cells come to market when it is not clear yet if it would be legally permissible in all European jurisdictions nor if it would be acceptable to a broad range of Type 1 Diabetes patients?

#### Informed Consent of Recipients

As asserted in our opening analysis of legal and ethical issues there is an obligation and challenge to obtain informed consent of recipients, but this is also morally essential. Recipients have the right to respect their autonomy and to have the opportunity to reject the treatment—and choose another—based on moral, religious, or any other system of beliefs. Ultimately the success (or failure) of hybrid beta-cell replacement therapies will hinge on if recipients find the product acceptable and will consent to its use.

However, the transition from medical research to clinical practice, also results in a change in the legally proscribed content of consent. In Europe, for medical research, the process and required information for consent is laid down explicitly and in detail in the Clinical Trials Regulations [59]. This means that all information must be “kept comprehensive, concise, clear, relevant, and understandable to a layperson [59].” However, no such consensus or legal standards have been agreed upon in the case of medical treatment.

Determining the content of consent may be challenging owing to the complexity of these hybrid therapies. Considerable uncertainties may also exist in some areas. For instance, long-term monitoring will be necessary to assess potential health risks of the use of highly manipulated and/or (genetically) modified xenogeneic or allogenic cells, such as transmission of zoonotic infections, epigenetic or genetic instability of the graft, or immunological or tumorigenic reactions in the recipient [52]. Long-term monitoring requires, at a minimum, a practical commitment for recipients, but from a psychological perspective, it suggests that there may be safety risks, which may be perceived as threatening. Furthermore, recipients will be required to relinquish some of the learned control they have developed over years of self-management regimes [60], which may cause relief but also anxiety at the idea that if something goes wrong with the hybrid product, they may not know or be able to influence this process. For example, systems with non-user-modifiable algorithms, which take full control of blood glucose, can be experienced by patients as resulting in a loss of autonomy [61]. The initial recipients are likely to be patients with poorly regulated diabetes and extensive secondary complications. These are patients with a perceived lack of alternative treatment options hoping for a cure, which could influence their decision making. If this influence amounts to coercion or interference with the exercise of their autonomy, it will be dependent upon individual patient circumstances.

While it is often assumed that a complete understanding of the technical and biological details of the product is required for informed consent, it is not clear whether “incomplete” understanding renders patients’ decisions to undergo treatment (or not) less autonomous. One study exploring the views of tissue engineers on relevant issues and goals of clinical trials with human tissue-engineered products suggests that participants may not always want to be informed in technical information about the composition of the product, but want to be informed mainly about issues that could directly affect their health status and quality of life [62]. However, we do not know, due to the lack of research in this area, what the needs and preferences are of Type 1 Diabetes patients with regards to consenting to receive a hybrid beta-cell replacement therapy.

Initially, there will be many uncertainties, and recipients will need to consider their own moral boundaries in balancing risks and potential benefits, and in envision their level of acceptance and psychological response to having a therapy implanted in their bodies, which is comprised of various cell sources. From an ethical and psychological perspective, clinicians are likely to have to go beyond what might be considered the minimum requirements of information to ensure that prospective recipients are appropriately informed and counselled to make their decisions.

### Access and Availability

Global accessibly and availability incorporate various areas that require attention including access among disadvantaged populations, recipient prioritisation and allocation of scarce resources.

#### Access Globally

Ethical, legal and psychosocial concerns arise regarding the potential limitations in accessibility due to the anticipated high costs associated with the clinical translation of regenerative therapies. High costs to develop the therapy will limit accessibility. If only those with financial means can benefit from the therapy, it may increase socioeconomic disparities at both local and global levels. However, over time, as the therapy becomes more established, costs are expected to decrease, potentially leading to costs-effectiveness [39]. For healthcare systems in the global South, where Type 1 Diabetes patients face already poor health outcomes and the production costs of insulin and insulin pumps are high [63, 64], it is expected that limited financial resources, laboratory facilities and specialized personnel required will hinder the manufacturing and administration of cell-based replacement therapies [8]. In addition, eventually these therapies for Type 1 Diabetes patients generated from donated cells could be patented. While patenting may promote innovation, quality control, and prevent misuse, it may also hinder open science and research, as well as equitable patient access to the therapy to patients [51].

#### Scarcity of Resources

In order to circumvent resource scarcity, reliable and ideally renewable source (e.g., allogenic stem cells) of insulin secreting beta-cells should be used to generate a hybrid beta-cell replacement therapy. Therefore, there are grounds to argue that funds should be channelled towards regenerative medicine solutions. However, there may be a concern that this allocation may divert resources away from other promising healthcare (technical) solutions for Type 1 Diabetes patients (e.g., hybrid-loop devices).

## Part 3—Practical Recommendations to Facilitate Translation

### Optimising Informed Consent

One practical solution to some of the challenges outlined regarding cell sourcing is to optimize the informed consent process. To guide this process and promote optimal informed consent, we propose the following recommendations:

First, language is important. There are different terms in circulation, ranging from bioartificial pancreas to cell-based products, some of which call into mind the *transplantation* of (bioartificial) *organs*, while others refer rather to medical devices (bio-*artificial*) or to advanced therapeutic medicinal products (*cell-based* therapy). Researchers, manufacturers and clinicians should be aware that terminology may affect patients’ perspectives on (the risks and potential benefits of) and understanding of hybrid beta-cell replacement therapy. Standardize nomenclature would help promote understanding for all parties involved.

Second, it may be difficult to understand the precise composition of a complex hybrid beta-cell replacement therapy and the implications of accepting it. Given this complexity and individual differences in the ability to understand information needs, strategies are needed to make information accessible and tailored. To support accessibility various modes of delivery can be used in addition to written, for example, diagrams, pictograms or visual timelines [65]. Scientific jargon should be avoided [66] and the required reading level should be no higher than high-school level. Tailoring of information can be achieved through a stepped approach, whereby a minimum of information is agreed upon with optional add-ons for those with greater information needs.

Third, as part of this minimum set of facts, patients should be informed as part of the informed consent process that removal of the bio-engineered pancreas (in its entirety) may not be possible [67].

Last, researchers and/or manufacturers should recognise that their responsibilities in relation to information provision go beyond that of obtaining informed consent from donors and recipients, and consider how they may effectively engage patient communities, donors and donor families and society at large, in discussions on cell-based replacement therapies for Type 1 Diabetes.

### Conduct Qualitative Research

A better understanding of patients’ perspectives on hybrid beta-cell replacement therapies will be crucial for the development of adequate informed consent processes. Qualitative research would be of added value to gain understanding of how patients needs and preferences can be met and under which conditions they would undergo treatment with a hybrid beta-cell replacement product. Insights from research on patients’ perspectives regarding hybrid beta-cell replacement therapies would help facilitate the clinical translation process. Patient representation through qualitative research will be necessary to ensure acceptance, uptake and adoption of such treatments.

### Public Policy

Effort should be dedicated to enhancing accessibility to ensure equitable distribution of the therapy. Moreover, to guarantee equal access to novel therapies, reimbursement policies will be necessary. These reimbursement decisions should not solely be based on clinical benefits, but also on patient’s preferences compared to alternative treatment options. Finally, regulations for cellular and gene therapies should be more globally harmonized.

## Conclusion

By utilizing an interdisciplinary approach to the analysis of the legal, ethical and psychosocial matters surrounding the translation of hybrid beta-cell replacement therapies, our group has not only identified unifying themes linking each discipline, but also revealed important next steps in resolving key barriers. While some of these issues have been navigated before in isolation, when combined, they become an unchartered territory, in particularly for patients and regulators. A comprehensive and interdisciplinary approach is required to guide hybrid beta-cell replacement therapy in the clinic in an acceptable and ethically sound manner. Researchers should collaborate across disciplinary fields and engage in dialogue, involving not only scientists but also patients, clinicians, citizens and policymakers. Patient engagement is particularly essential in this clinical translation process to ensure the acceptance, uptake and adoption of such treatments in routine clinical practice.

## VANGUARD Consortium Partners

Ekaterine Berishvili, Laura Mar Fonseca, Fanny Lebreton, Kevin Bellofatto, Juliette Bignard (Department of Surgery, University of Geneva, Geneva, Switzerland); Jochen Seissler, Leila Wolf-van Buerck, Mohsen Honarpisheh, Yichen Zhang, Yutian Lei, Monika Pehl (Diabetes Centre—Campus Innenstadt, Medizinische Klinik und Poliklinik IV, Klinikum der Ludwig-Maximilians-Universität München, Germany); Antonia Follenzi, Christina Olgasi, Alessia Cucci, Chiara Borsotti, Simone Assanelli (Department of Health Sciences, University of Piemonte Orientale, Novara, Italy); Lorenzo Piemonti, Antonio Citro, Silvia Pellegrini, Cataldo Pignatelli, Francesco Campo (IRCCS Ospedale San Raffaele, Diabetes Research Institute, Milano, Italy); Olivier Thaunat (Department of Transplantation, Nephrology and Clinical Immunology, Lyon Claude Bernard University, Lyon, France); Devi Mey, Chiara Parisotto, Giovanna Rossi (European Society for Organ Transplantation, Padova, Italy); Patrick Kugelmeier, Petra Wolint, Markus Mühlemann, Karolina Pal-Kutas (Kugelmeiers AG, Erlenbach, Switzerland); Marco Cavallaro, Julia Götz, Jeanette Müller (Accelopment Switzerland Ltd.).
